# Albumin Enhances Microvascular Reactivity in Sepsis: Insights from Near-Infrared Spectroscopy and Vascular Occlusion Testing

**DOI:** 10.3390/jcm14144982

**Published:** 2025-07-14

**Authors:** Rachael Cusack, Alejandro Rodríguez, Ben Cantan, Orsolya Miskolci, Elizabeth Connolly, Gabor Zilahi, John Davis Coakley, Ignacio Martin-Loeches

**Affiliations:** 1 Department of Intensive Care Medicine, Multidisciplinary Intensive Care Research Organization (MICRO), St. James’ Hospital, D08 NHY1 Dublin, Ireland; 2 School of Medicine, Trinity College Dublin, D02 PN40 Dublin, Ireland; 3 Hospital Universitario Joan XXIII de Tarragona, Rovira & Virgili University, Centre for Biomedical Research Network Respiratory Diseases (CIBERES), 43005 Tarragona, Spain

**Keywords:** albumin, colloid, critical care, fluid resuscitation, intensive care, microcirculation, randomized controlled trial, sepsis, septic shock

## Abstract

**Background/Objectives**: In septic shock, microcirculatory dysfunction contributes to organ failure and mortality. While sidestream dark-field (SDF) imaging is the reference method for assessing microvascular perfusion, its complexity limits routine use. This study evaluates near-infrared spectroscopy (NIRS) with vascular occlusion testing (VOT) as a potential bedside tool for monitoring microcirculatory changes following fluid resuscitation. **Methods**: Sixty-three fluid-responsive patients with sepsis were randomized to receive either 20% albumin or crystalloid. NIRS-VOT and sublingual SDF measurements were obtained at baseline and 60 min post-resuscitation. The reoxygenation slope (ReOx) derived from NIRS was calculated and compared with clinical severity scores and SDF-derived microcirculatory parameters. **Results**: ReOx significantly increased from baseline to 60 min in the albumin group (*p* = 0.025), but not in the crystalloid group. However, between-group differences at 60 min were not statistically significant. ReOx at 60 min was inversely correlated with APACHE II score (ρ = −0.325) and lactate (ρ = −0.277) and showed a weak inverse trend with norepinephrine dose. AUROC for ICU survival based on ReOx was 0.616. NIRS ReOx showed weak correlations with SDF parameters, including the number of crossings (*p* = 0.03) and the consensus proportion of perfused vessels (CPPV; *p* = 0.004). **Conclusions**: NIRS-VOT detected microcirculatory trends after albumin administration but showed limited agreement with SDF imaging. These findings suggest that NIRS and SDF assess different physiological domains. Further studies are warranted to define the clinical utility of NIRS as a microcirculation monitoring tool (Clinicaltrials.gov: NCT05357339).

## 1. Introduction

Microcirculation is the final effector site of the cardiovascular system, facilitating the exchange of oxygen and metabolites at the capillary level [1]. In sepsis, this intricate network becomes profoundly dysregulated, with decreased functional capillary density and increased flow heterogeneity contributing to tissue hypoxia and organ dysfunction [2,3]. While fluid resuscitation is a cornerstone of early sepsis management, conventional hemodynamic targets often fail to reflect the true state of the microvasculature, leading to a disconnect between macrocirculation and tissue perfusion [4]. Albumin, with its endothelial-stabilizing properties and ability to maintain oncotic pressure, has been suggested as superior to crystalloids in restoring microvascular flow in septic shock [5,6,7].

Stroboscopic LED ring-based imaging, such as sidestream dark-field (SDF) imaging, is currently considered the gold standard for bedside visualization of the microcirculation, and has been used to demonstrate microvascular recruitment and improved vessel density following albumin resuscitation [8]. However, SDF’s limited availability, cost, and need for specialized expertise restrict its widespread clinical use [9,10]. Near-infrared spectroscopy (NIRS), particularly when combined with a vascular occlusion test (VOT), offers a non-invasive, operator-independent alternative. It provides real-time insight into tissue oxygen dynamics by measuring regional saturation (rSO_2_) and evaluating deoxygenation and reoxygenation slopes during ischemic challenges [11,12,13]. Despite its promise, NIRS has not yet been validated against established microcirculatory tools such as SDF in septic patients.

This study aims to explore the feasibility of using NIRS as a surrogate for direct microcirculatory assessment. We build upon our previous findings, which showed that 20% albumin significantly improves microvascular density and activity as measured by SDF microscopy [14]. In this study, we focus on an NIRS-based assessment of tissue oxygenation and microvascular reactivity, comparing albumin to crystalloid resuscitation. Our hypothesis is that albumin will result in superior improvements in NIRS-derived parameters—specifically steeper reoxygenation and deoxygenation slopes—reflecting enhanced microvascular responsiveness. Furthermore, we aim to evaluate the correlation between these NIRS metrics and previously reported SDF parameters to determine whether NIRS can serve as a reliable, practical tool for assessing microcirculation in critically ill patients.

## 2. Materials and Methods

### 2.1. Study Design and Setting

This observational study was nested within the MICALB21 trial (ClinicalTrials.gov ID: NCT05357339), which investigated microcirculatory responses to fluid resuscitation in sepsis and septic shock. Recruitment occurred in the mixed medical–surgical intensive care unit (ICU) at St. James’s Hospital, Dublin, between September 2021 and July 2023. Adult patients (≥18 years) who met Sepsis-3 diagnostic criteria and were undergoing planned sublingual microcirculatory assessment were eligible.

This study received ethical approval from the St James’s Hospital and Tallaght University Hospital Research Ethics Committee (SJH/TUH REC; approval number 2019-11-269040 5642). Given the critically ill status of participants, familial assent and deferred consent were obtained in accordance with Health Research Consent Declaration Committee (HRCDC) guidelines (reference ID: 20-031-AF1). In cases where assent was withdrawn after inclusion, patient data were deleted in line with HRCDC standards. Full informed consent was obtained from patients following recovery and ICU discharge.

Patients received standard ICU management, and all microcirculation measurements were non-invasive and did not interfere with clinical care. Fluid responsiveness was assessed using pulse pressure variation (PPV). Patients were randomized to receive either 20% albumin (intervention group) or crystalloid (control group) in 100 mL boluses, over 15 min, until the PPV measured <12%. Microcirculatory monitoring was performed at baseline (T0), 15 min (T15), and 60 min (T60) after fluid bolus, using two complementary technologies: near-infrared spectroscopy (NIRS) with vascular occlusion testing (VOT) and sidestream dark-field (SDF) imaging.

### 2.2. Near-Infrared Spectroscopy (NIRS) and Vascular Occlusion Testing (VOT)

NIRS-VOT was performed using the INVOS 5100c oximeter (Medtronic, Minneapolis, MN, USA) with disposable adult SomaSensor probes (SAFB-SM). The device uses reflectance-mode near-infrared light to measure regional oxygen saturation (rSO_2_), capturing signals from blood vessels smaller than 1 mm. Probes were applied to the skin after acetone cleansing.

For the VOT, a sphygmomanometer cuff was inflated to 200 mmHg around the upper arm. After rSO_2_ stabilization (<2% variation over 30 s), ischemia was induced for 5 min. Upon cuff release, the reoxygenation phase was recorded. The following parameters were derived:

- Deoxygenation slope (DeOx): rate of rSO_2_ decline during occlusion.
- Reoxygenation slope (ReOx): rate of rSO_2_ recovery post-occlusion test, capturing both early (12 s) and total reoxygenation.

Measurements were obtained at T0, T15, and T60. Signals were continuously recorded and exported as .csv files for a blinded analysis.

### 2.3. Sidestream Dark-Field (SDF) Imaging

SDF imaging was conducted using the Microscan device (Microvision Medical, Amsterdam, The Netherlands), the gold standard for bedside microvascular visualization. Green light (540 nm) illuminated red blood cells in the sublingual microvasculature. Measurements were taken at the same time points as NIRS (T0, T15, and T60). Saliva was removed prior to image acquisition. Videos were analyzed using AVA 4.3 software, extracting

- De Backer density;
- Total vessel density;
- Perfused vessel density;
- Proportion of perfused vessels.

### 2.4. Outcomes

The primary outcome was the change in ReOx over time (T0, T15, and T60), comparing albumin and crystalloid groups.

Secondary outcomes included

- Correlations between NIRS-derived ReOx/DeOx and SDF parameters (e.g., De Backer density and perfused vessel proportion);
- Differences in baseline NIRS-VOT response between fluid groups;
- Influence of baseline microcirculatory status on physiological responses to fluid type.

These outcomes were designed to assess both the physiological response to resuscitation and the potential of NIRS to serve as a surrogate for SDF in monitoring microcirculation.

### 2.5. Statistical Analysis

All statistical analyses were conducted using R (RStudio Version 2025.05.0+496). Group comparisons were performed using the Wilcoxon rank-sum and Mann–Whitney U tests. Correlations between NIRS and SDF parameters were assessed using Pearson and Spearman coefficients. To assess the degree of agreement between each device in measuring changes in microcirculation, we also tested intra-class correlations (ICCs). This quantifies the similarity of readings from each device within clusters.

Linear regression models evaluated predictive associations. Correlation matrices were generated for visualization. Significance was defined as *p* < 0.05.

SDF metrics were analyzed using AVA 4.3 software, and NIRS signals were processed from exported .csv files. A single VOT was performed per subject at each time point. Image and data analyses were performed blinded to group allocation.

## 3. Results

### 3.1. Patient Characteristics

A total of 63 patients with sepsis or septic shock were included in the analysis. Twenty-one patients (33%) were female. The mean age of the cohort was 59 years (range 19–86). Baseline clinical characteristics, including APACHE II and SOFA scores, were similar between the albumin and crystalloid groups (Table 1).

### 3.2. Resuscitation

The albumin group received a mean volume of 301.3 mL of 20% albumin, and the control group received a bolus volume of 373.4 mL (t = 1.6, *p*-value = 0.19). Starting hemoglobin in the albumin group was 10.2 g/dL, and in the control group, it was 11.2 g/dL (t = 1.67, *p*-value = 0.11). There was no significant change in hemoglobin level following albumin resuscitation (10.2 g/dL vs. 9.7 g/dL, *p*-value = 0.15), but there was a reduction from 11.2 g/dL to 9.4 g/dL (*p*-value = 0.001) in the group receiving crystalloid.

### 3.3. Near-Infrared Spectroscopy: Reoxygenation and Deoxygenation Slopes

**Figure 1:**
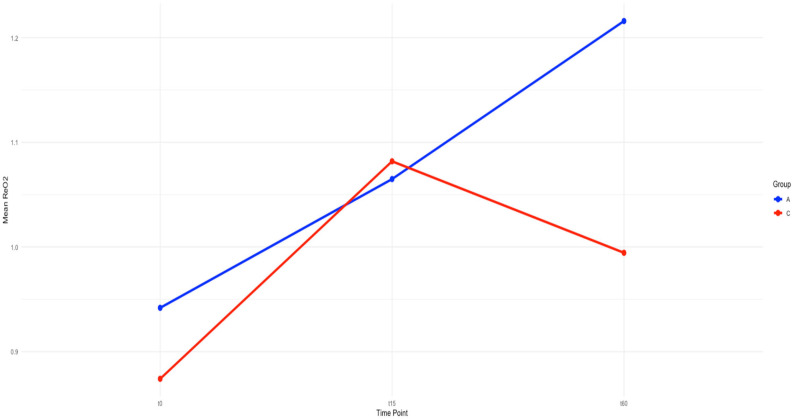
Mean change in reoxygenation slope from time 0 to time 15 and time 60 by group. Group A in blue denotes the albumin group, and group C, in red, denotes the control group. The x-axis signifies time, specifically at the three measured time points of pre-resuscitation, 15 min after resuscitation, and one hour post-resuscitation. The y-axis signifies the mean reoxygenation slope in each group. Reoxygenation slopes at time 0: albumin, 0.942 [95% CI 0.6338–1.284]; control, 0.874 [95%CI 0.682–1.08], *p*-value = 0.729. The change from time 0 to time 60 in the albumin group was t = 1.7, *p*-value = 0.0249, and in the control group, it was t = 1.69, *p*-value = 0.156. The baselines are not different; however, following albumin resuscitation, the reoxygenation slope at time 60 is 1.215, whereas in the control group, it is 0.994, t = 0.933, *p*-value = 0.35.

At baseline (T0), reoxygenation slope (ReOx) values were similar between groups (Table 1, Figure 1). In the albumin group, a statistically significant increase in ReOx was observed between baseline and 60 min (*p* = 0.025). In the crystalloid group, no significant change in ReOx was detected over the same period (*p* = 0.156). The absolute difference in ReOx at 60 min between the albumin and crystalloid groups was not statistically significant (mean ReOx 1.215 vs. 0.994; *p* = 0.35; Table 1, Figure 1).

**Figure 2:**
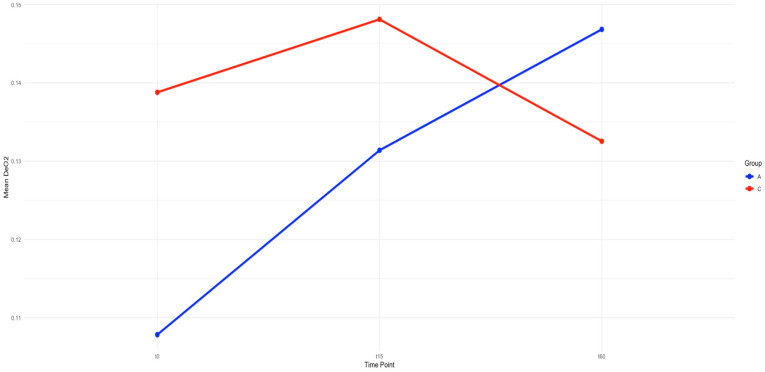
Mean change in deoxygenation slope from time 0 to time 15 and time 60 by group. Group A in blue denotes the albumin group, and group C, in red, denotes the control group. The x-axis signifies time, specifically at the three measured time points of pre-resuscitation, 15 min after resuscitation, and one hour post-resuscitation. The y-axis signifies the mean deoxygenation slope in each group. Although the baselines are different, there is consistent improvement in the deoxygenation slope in the albumin group from pre-resuscitation to post-resuscitation, with this improvement being maintained to 60 min post-fluid bolus.

Deoxygenation slopes (DeOx) did not differ significantly between the groups at any time point (Table 1, Figure 2). In the albumin group, a directional increase in DeOx was observed post-resuscitation compared to baseline, consistent with a pattern of improvement.

### 3.4. Association Between NIRS Reoxygenation Slopes and Clinical Outcomes

**Figure 3:**
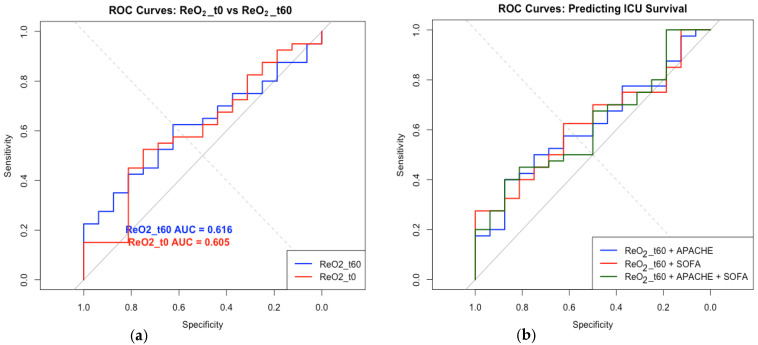

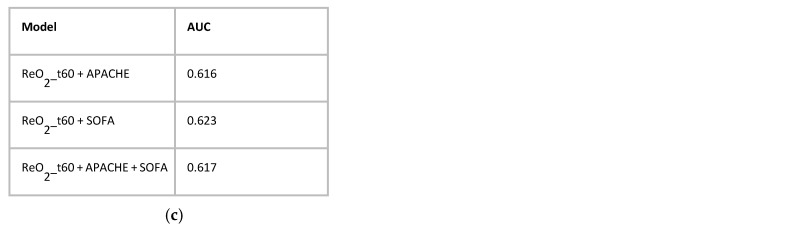
(**a**) Comparison of ROC curves for reoxygenation at time 0 vs. reoxygenation at time 60 on the prediction of ICU survival. In red, the reoxygenation slope at time 0 pre-resuscitation shows the ability to predict ICU survival at an area under the curve value of 0.605, whereas in blue, at 60 min post-resuscitation, the reoxygenation slope shows minimal improvement in ICU survival prediction, with an area under the curve value of 0.616. (**b**) Comparison of multivariable analysis combining reoxygenation with other predictors such as APACHE and SOFA. Combining the reoxygenation slope at 60 min post-resuscitation with the APACHE score (in blue), the SOFA score (in red), or both together (in green) had limited ability in improving the prediction of ICU survival. (**c**) AUC values denoting the curves in (**b**) combining reoxygenation slope at time 60 with APACHE, SOFA, and APACHE plus SOFA.

The reoxygenation slope at 60 min was associated with an area under the curve (AUC) of 0.616 for ICU survival. A cutoff value of 0.973 was identified using the Youden index, yielding 62.5% sensitivity and specificity (Figure 3). The addition of ReOx at 60 min to multivariable models containing APACHE II and SOFA scores did not improve the model’s discriminatory ability (AUROC range: 0.616–0.623).

**Figure A1:**
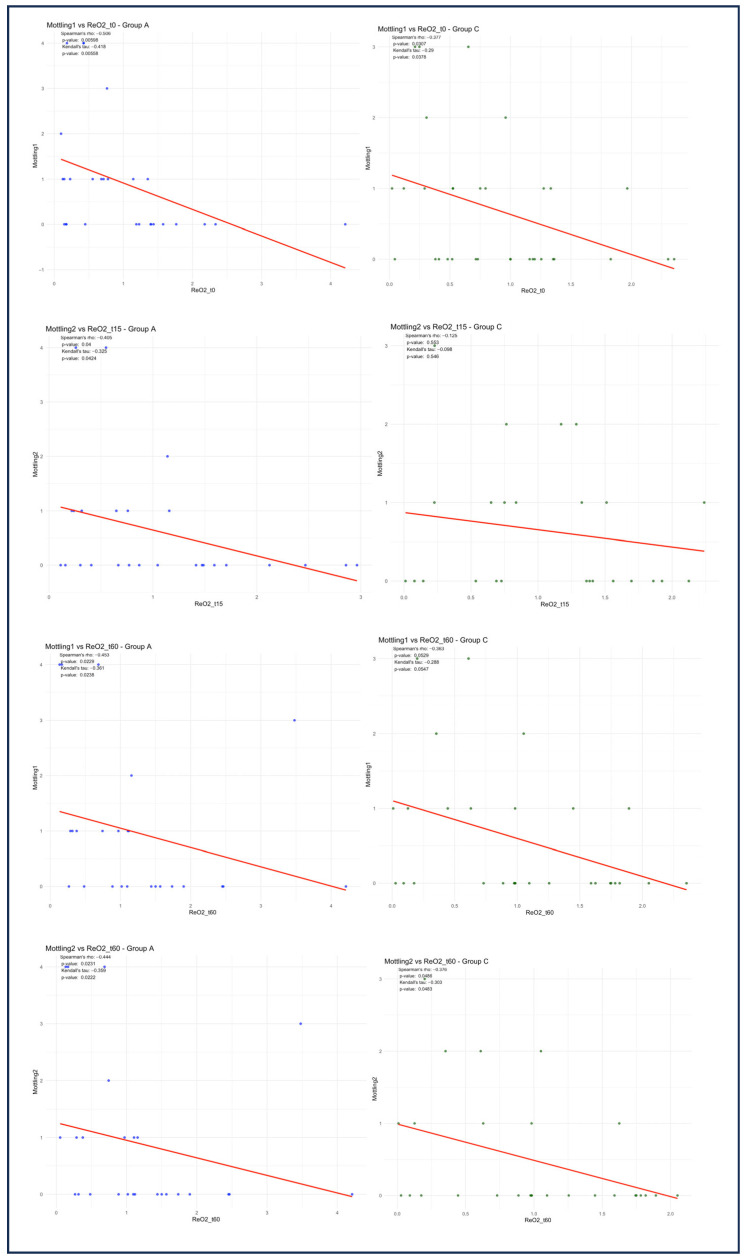
Linear regression scatterplot diagrams depicting relationships between reoxygenation slopes and mottling score.

**Figure A2:**
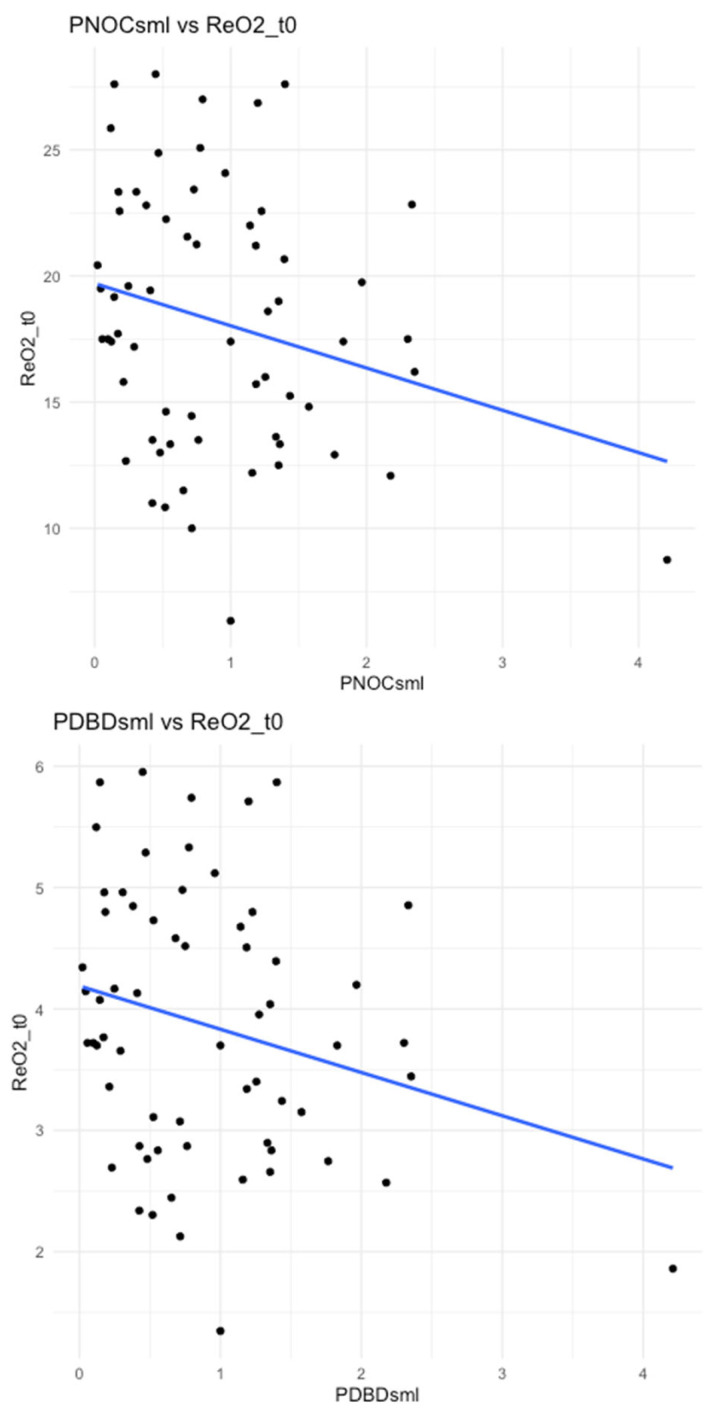
Linear regression scatterplots with fitted line showing the correlation between perfused number of crossings (small) and reoxygenation slope at time 0 (*ρ* = 0.26, *p* = 0.05).

No significant association was observed between reoxygenation slopes and total ICU or hospital length of stay (LOS) (ReOx T0 vs. hospital LOS: r = 0.09, *p* = 0.53; ReOx T60 vs. hospital LOS: r = 0.11, *p* = 0.42; Appendix A Figure A1 and Figure A2). When dichotomized into ICU stays longer than 7 days, the combined use of ReOx at baseline and 60 min yielded an AUROC of 0.638.

The ReOx slope at 60 min showed a negative correlation with norepinephrine dose (Spearman r = −0.229, *p* = 0.08). No significant association was found with vasopressor duration.

At 60 min, ReOx was inversely correlated with APACHE II score (Spearman r = −0.325, *p* = 0.01). Correlations with SOFA scores were not statistically significant. A negative correlation was observed between ReOx at 60 min and lactate (r = −0.277, *p* = 0.04). No correlation was found between ReOx and CRP, WCC, or ScvO_2_.

ReOx at time 0 was significantly correlated with mottling scores at T0 and T15, and ReOx slope at T60 was correlated with mottling at T0 and T15, but not at T60. When analyzed by treatment group, significant correlations persisted at T0 and T15 for both albumin and control, although only the albumin group maintained significance at T15, suggesting that the relationship between NIRS VOT and mottling is strongest early in resuscitation and diminishes over time, with no significant differences found between the two groups (Appendix A Figure A1).

### 3.5. Correlation Between NIRS and Sidestream Dark-Field Imaging

**Figure 4:**
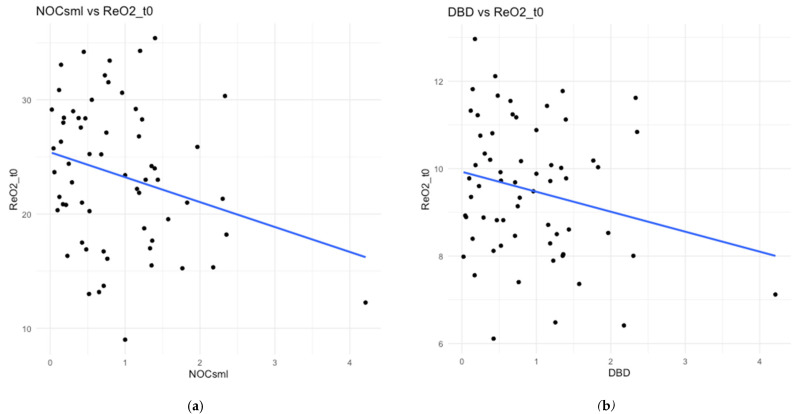
Linear regression scatterplots showing correlation of microcirculation metrics of (**a**) number of crossings <20 μm and (**b**) De Backer density at time 0, with reoxygenation slope at time 0 (*ρ* = 0.26, *p* = 0.03, *ρ* = 0.23, and *p* = 0.07).

Microcirculation preceding fluid resuscitation was weakly correlated with ReOx at T0, but only significant for the number of crossings (small, i.e., <20 μm) and the perfused number of crossings (small) (*ρ* = 0.26, *p* = 0.03, Figure 4; *ρ* = 0.024, *p* = 0.05, Appendix A Figure A2).

At T60, weak correlations were observed between ReOx slopes and SDF-derived metrics, De Backer density (*ρ* = −0.28, *p* = 0.03), the number of crossings (*ρ* = −0.28, *p* = 0.03), and the consensus proportion of perfused vessels (CPPV). The slope between ReOx and CPPV was 0.24 (*p* = 0.07), and for CPPV (small, i.e., <20 μm), it was 0.26 (*p* = 0.05).

Multivariable linear regression with time as a categorical variable and ten microcirculation parameters identified only CPPV as a significant predictor of reoxygenation slope at 60 min (*p* = 0.004). The model explained 9% of the variation in ReOx (adjusted R*^2^* = 0.089).

### 3.6. Reliability and Change over Time

Intra-class correlation coefficients (ICCs) across time points were <0.2 for ReOx and other microcirculation variables. When analyzed using a fixed-rater (ICC3k) model, statistically significant consistency was found for all SDF-derived parameters (Table 2). Changes in ReOx between baseline and 60 min were not significantly correlated with changes in SDF parameters over the same time interval.

## 4. Discussion

This study is the first to evaluate near-infrared spectroscopy (NIRS) with vascular occlusion testing (VOT) as a bedside tool to assess microcirculation in patients with sepsis, using sidestream dark-field (SDF) imaging as the reference method. Lower NIRS-derived reoxygenation slopes were associated with higher illness severity, correlating modestly with APACHE II scores, lactate levels, mottling, vasopressor requirements, and ICU length of stay. However, ReOx alone was not a strong predictor of ICU or hospital mortality, and its inclusion in multivariable models did not improve outcome discrimination.

Following resuscitation, the albumin group demonstrated a statistically significant improvement in reoxygenation slope between baseline and 60 min. These findings are consistent with our previously published data from the same cohort, which demonstrated that a 20% albumin bolus significantly improved SDF-derived microvascular density and activity at 15 and 60 min compared to no change in the crystalloid group (*p* < 0.005). In that analysis, both groups were fluid-responsive and well-matched for APACHE and SOFA scores [14]. The current NIRS-based analysis partially mirrors these trends, though without consistent statistical significance across all measures.

The weak correlation between ReOx and SDF-derived metrics such as De Backer density and the consensus proportion of perfused vessels (CPPV) raises concerns regarding the sensitivity of NIRS in capturing discrete microcirculatory changes. While these measures of absolute microcirculation density and function showed a statistically significant association with ReOx, the overall explanatory power was limited. These results suggest that NIRS, though useful for indicating broad trends in tissue oxygenation, may lack the resolution to detect microvascular improvements with the same fidelity as SDF. The weak correlation observed in this study also raises important methodological questions. While ReOx increased after albumin administration, consistent with microvascular improvements captured by SDF, the statistical correlation between the two modalities remained modest. These findings suggest that NIRS-VOT and SDF may be measuring related but distinct physiological domains.

In our study, NIRS-VOT was performed on the thenar eminence, a superficial site with high metabolism. However, anatomical challenges such as edema, limb dressings, or intravascular access devices limited usability in approximately one-third of patients included in the original MICALB study (n = 100). Resting tissue oxygenation parameters are insensitive to microvascular changes; however, the use of a vascular occlusion test has been found to increase the usability of this device to detect microvascular reactivity in response to hypovolemia, hemorrhage, and septic shock [15,16]. Previous studies have found it to be comparable to devices designed for use at the thenar eminence, making it a more widely applicable device that can be used not only on the brain, but also on the kidney, gut, and peripheral muscle [17,18,19,20]. These studies endorse the versatility of the NIRS and VOT in clinical practice.

The INVOS 5100c system used in this study was selected based on its widespread availability and prior use in both cerebral and peripheral monitoring [17,18,21]. Though initially designed for cerebral oximetry, its application in dynamic VOT protocols has been reported in settings such as trauma, hemorrhage, and sepsis [15,22,23,24]. During vascular occlusion, reoxygenation and deoxygenation slopes offer dynamic insights into peripheral oxygen kinetics. Despite differences in absolute rSO_2_ values across devices and sensors, previous work has demonstrated consistency in slope trends [17,23]. Studies comparing devices have found that they can each behave differently, and different algorithms may provide differences in responsiveness and readings by reducing the signal-to-noise ratio [25,26].

Given that our prior analysis showed clear SDF-detected improvements in microcirculation after albumin (with statistically significant increases in perfused vessel density and microvascular flow), the lack of strong parallel findings with NIRS leads to doubt over whether NIRS-VOT, in its current form, is sufficiently accurate to detect clinically meaningful changes in microcirculation [27,28,29]. NIRS may provide indirect insights into oxygen delivery and consumption, but it does not appear to fully capture the structural and functional changes observed with SDF imaging.

Despite limitations in sensitivity, near-infrared spectroscopy with vascular occlusion testing (NIRS-VOT) presents several practical advantages. It is non-invasive, repeatable, and does not require calibration. Compared to sidestream dark-field (SDF) imaging, NIRS-VOT requires less training, is easier to perform, and is already available in many ICUs as part of routine cerebral or somatic oxygenation monitoring equipment [30,31,32,33]. These characteristics make it a feasible tool for bedside microcirculation assessment in critically ill patients.

Importantly, this study supports the use of dynamic rather than static NIRS measurements. Static tissue oxygen saturation values (rSO_2_) have shown inconsistent correlation with microcirculatory function and are influenced by local tissue properties and sensor variation. In our analysis, static rSO_2_ values were less informative than VOT-derived parameters such as the reoxygenation slope (ReOx), which correlated with APACHE II scores and lactate. Dynamic changes during occlusion and reperfusion provide functional insight into oxygen delivery, consumption, and microvascular responsiveness, offering more relevant information for critically ill patients undergoing resuscitation [33,34].

Previous studies have explored the prognostic role of static rSO_2_ values. In a prospective observational study, Rodriguez et al. (2011) demonstrated that lower brachioradialis muscle rSO_2_ was associated with increased mortality in septic shock patients, with non-survivors consistently exhibiting rSO_2_ ≤ 60% over the first 24 h [35]. Although static values may help stratify risk at admission, they do not appear sensitive enough to detect microvascular improvement over time or guide response to therapy, which are roles better suited to dynamic assessments like NIRS-VOT.

In clinical practice, the ability to monitor microvascular responsiveness dynamically and non-invasively could enhance the individualization of fluid therapy and other interventions. Our findings suggest that ReOx could serve as a surrogate marker of peripheral perfusion status and potentially guide treatment titration. However, ReOx alone was not sufficient to improve multivariable models predicting outcomes, highlighting the need for integration with broader clinical and biochemical assessments.

Overall, the feasibility, availability, and physiological insight offered by NIRS-VOT support its further investigation as a complementary tool for tracking microcirculatory trends in septic shock, particularly where SDF is unavailable.

This study’s strengths include its prospective design, comparison of two fluid types, parallel SDF imaging as a reference standard, and the use of dynamic NIRS-VOT parameters rather than static rSO_2_ values. It represents the first clinical trial to systematically compare these two technologies in septic patients undergoing fluid resuscitation.

Limitations include the relatively small sample size and missing data due to anatomical or device-related constraints. The INVOS device was not specifically designed for microvascular monitoring, and tissue edema may have impaired measurement accuracy. Despite careful site preparation and probe placement, variability in skin and muscle characteristics likely influenced results. Importantly, although trends in ReOx improved after albumin and aligned with SDF findings, the modest correlation between modalities suggests that NIRS should not yet be considered equivalent to SDF for precise microcirculation assessment.

## 5. Conclusions

NIRS monitoring with dynamic VOT is a feasible bedside method for tracking peripheral microcirculatory response in septic patients. In this cohort, NIRS-derived reoxygenation slopes reflected clinical severity and treatment response trends but demonstrated only limited correlation with SDF-derived microvascular metrics. These findings raise questions about the sensitivity of NIRS in detecting microcirculation changes and highlight the need for further investigation. Larger studies are warranted to determine whether NIRS-VOT can support individualized therapy and complement more established imaging techniques in the management of septic shock.

## Figures and Tables

**Table 1:** Summary of characteristics of final patient cohort.

	Control	Albumin
Survived ICU (n)	28	18
ICU LOS [mean (min–max)]	24 (3–122)	19 (2–190)
Antibiotic LOT [mean (min–max)]	15 (3–49)	18 (2–119)
Mechanical ventilation days [mean (min–max)]	12 (1–81)	8 (2–26)
Hospital LOS days [mean (min–max)]	53 (4–183)	38 (3–195)
Vasopressor days [mean (min–max)]	6 (1–29)	7 (1–34)
APACHE [mean (min–max)]	30 (7–42)	27 (7–43)
SOFA [mean (min–max)]	10 (3–17)	9 (1–17)
Age [mean (min–max)]	58 (24–85)	56 (19–86)
Norad mcg/kg/min [mean (min–max)]	0.204 (0–0.8)	0.193 (0–0.36)
Lactate mmol/L [mean (min–max)]	3.4 (0.8–13.5)	2.4 (1.1–5.6)
WCC × 10^9^/L [mean (min–max)]	15.6 (0.4–45.6)	13.9 (0.1–27)
CRP mmol/L [mean (min–max)]	162 (1.2–412)	118 (3.2–370)
ScVo2% [mean (min–max)]	72.4 (51.7–84.9)	72.5 (42.9–88)
RRT	3	4
Reoxygenation slope time 0 [mean (95% CI)]	0.874(0.68–1.08)	0.94 (0.63–1.28)
Reoxygenation slope time 15 [mean (95% CI)]	1.08 (0.83–1.33)	1.07 (0.74–1.39)
Reoxygenation slope time 60 [mean (95% CI)]	0.99 (0.74–1.25)	1.22 (0.809–1.62)
Deoxygenation slope time 0 [mean (95% CI)]	0.14 (0.12–0.16)	0.11 (0.06–0.16)
Deoxygenation slope time 15 [mean (95% CI)]	0.15 (0.12–0.17)	0.14 (0.11–0.16)
Deoxygenation slope time 60 [mean (95% CI)]	0.14 (0.11–0.16)	0.15 (0.12–0.18)

**Table 2:** Intra-class correlations for reoxygenation slope and each microcirculation variable measured by the Microscan SDF camera (Microvision Medical, Netherlands).

Variable	ICC	*p*-Value
NOC	0.62	1.56 × 10^−8^
DBD	0.48	0.00013
NOCsml	0.47	0.00018
DBDsml	0.38	0.004
PNOC	0.61	6.45 × 10^−8^
PDBD	0.51	2.54 × 10^−5^
PNOCsml	0.46	0.0004
PDBDsml	0.40	0.0018
CPPV	0.61	2.12 × 10^−8^
CPPVsml	0.57	1.61 × 10^−6^

**Figure A3:**
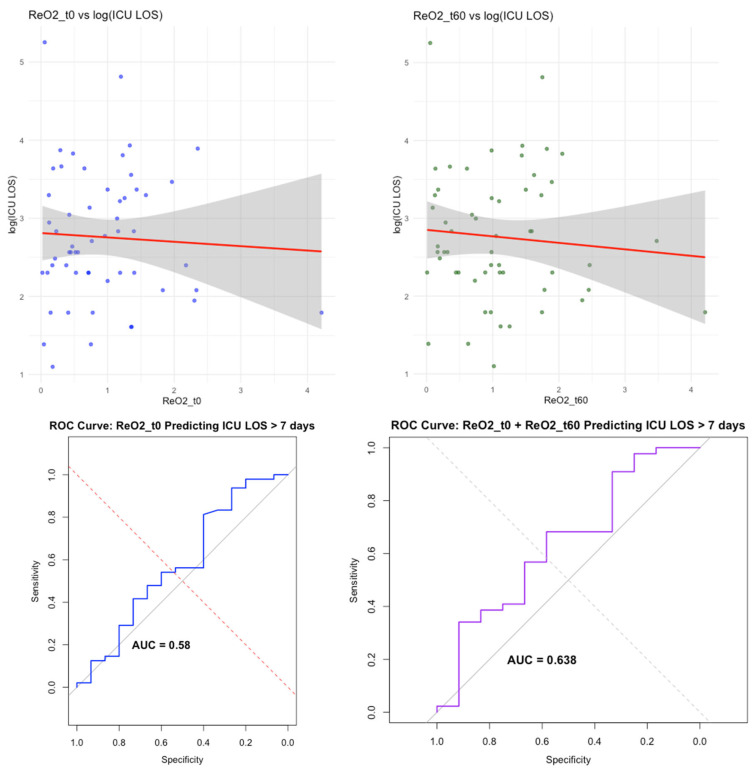
Linear regression and ROC curves showing poor association between reoxygenation and ICU length of stay.

**Figure A4:**
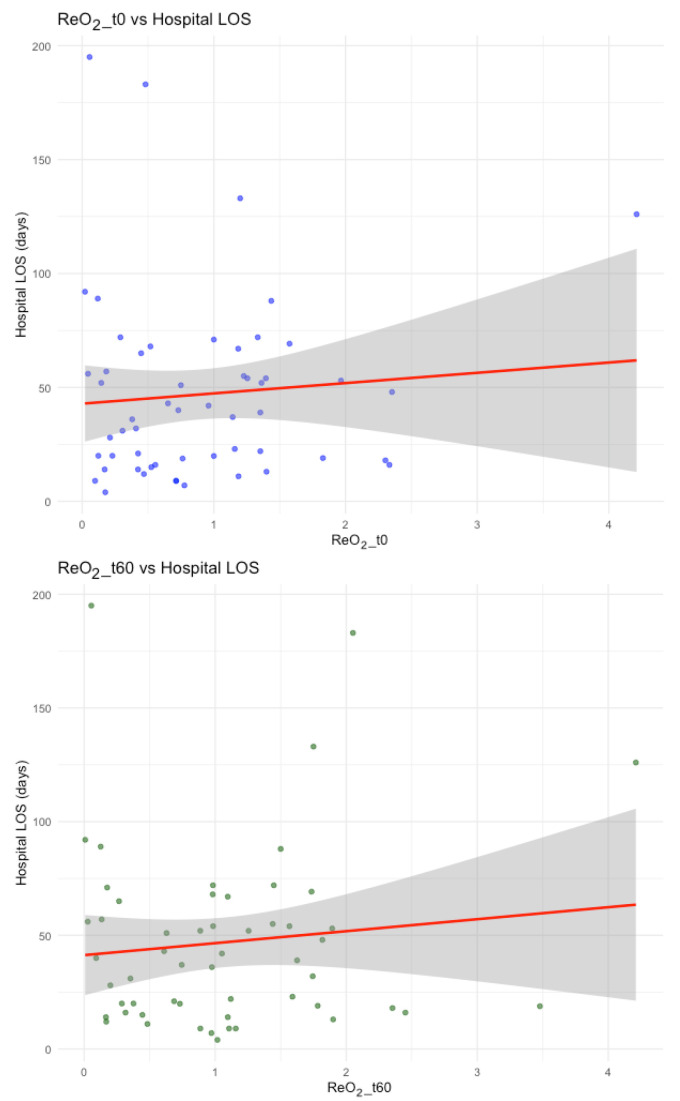
Linear regression scatterplots charting the correlation between reoxygenation slope and hospital length of stay. The colors are reoxygention slopes for each case.

**Figure A5:**
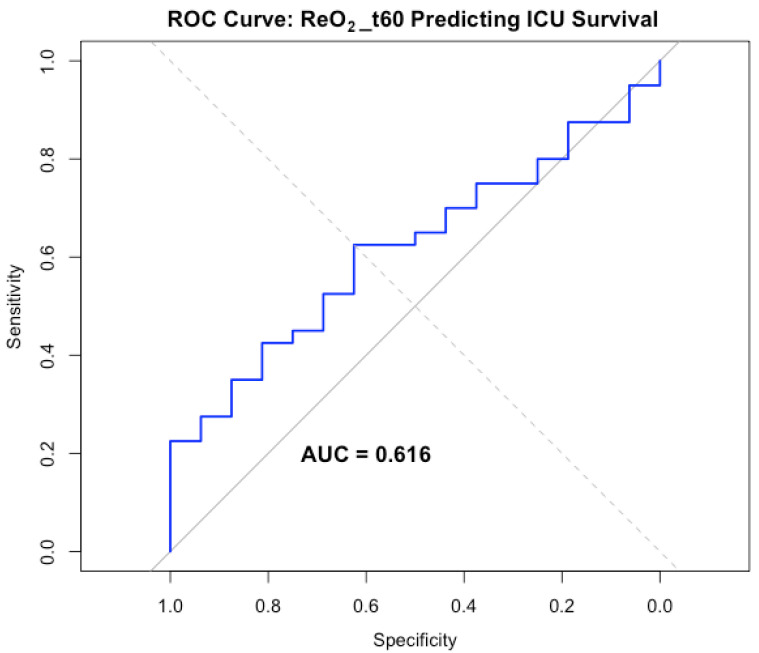

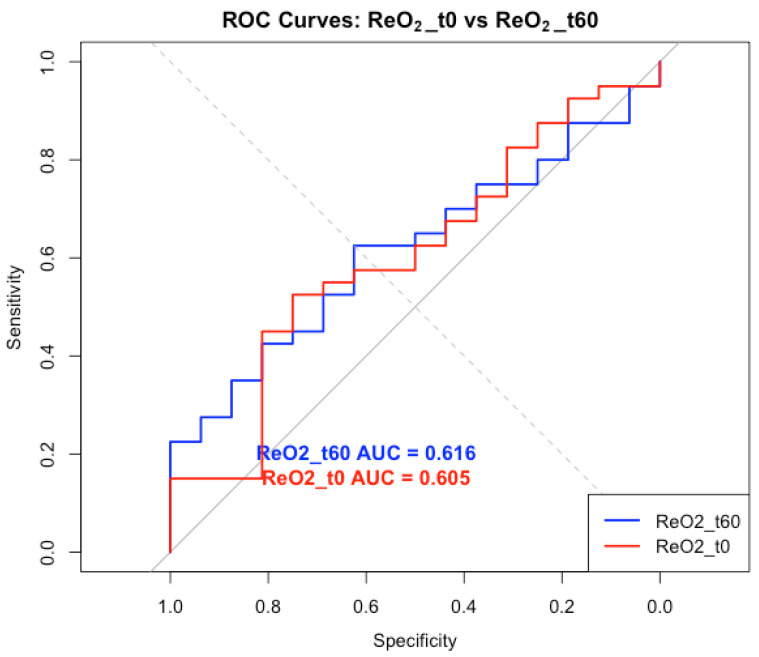
ROC curves of reoxygenation slopes and ICU survival.

## Data Availability

The datasets generated and analyzed during the current study are available from the corresponding author upon reasonable request. Data sharing complies with international, institutional, and ethical guidelines.

## Abbreviations

The following abbreviations are used in this manuscript:

SDF	Sidestream dark-field
NIRS	Near-infrared spectroscopy
VOT	Vascular occlusion test
ReOx	Reoxygenation slope

## Appendix A

### Appendix A.1. NIRS and Mottling Score

Mottling scores are used in clinical practice as bedside indicators of reduced microcirculation. This score, as well as its variation during resuscitation, is associated with 14-day mortality in septic shock patients [36]. We correlated mottling scores throughout the resuscitation of our cohort with NIRS VOT. We found that ReOx at time 0, preceding resuscitation, was significantly correlated with mottling score at time 0 (*ρ* = −0.432, *p* = 0.0005) and 15 min (*ρ* = −0.411, *p* = 0.001) following resuscitation. ReOx slope at one hour after resuscitation was also correlated with mottling score at time 0 (*ρ* = −0.392, *p* = 0.0034) and time 15 (*ρ* = −0.394, *p* = 0.0032). There were no significant correlations between mottling at 60 min and any ReOx slope, or with ReOx at 15 min and any mottling score. However, there were trends at ReOx at 15 min and mottling at times 1 and 15 min (*p* = 0.07, *p* = 0.06), indicating a signal warranting further investigation.

Looking at the groups of albumin and control, we found significant correlations between ReOx slopes at time 0 with mottling scores at time 0 and 15 min for the albumin group (*ρ* = −0.506, *p* = 0.006, *ρ* = −0.426, *p* = 0.02) and the control (*ρ* = −0.377, *p* = 0.031, *ρ* = −0.408, *p* = 0.02). At 15 min, this trends falters and the albumin group remains *ρ* significantly correlated between mottling scores and NIRS VOT (*ρ*= −0.444, *p* = 0.02, *ρ* = −0.405, *p* = 0.04), but the control group is not (*ρ* = −0.08, *p* = 0.72, *ρ* = −0.125, *p* = 0.5). However, the mottling score at time 3, 60 min after resuscitation, is not strongly associated with any ReOx slope. This suggests that the relationship is strong in the early stages of resuscitation but falls away later. Further study into the relationship of NIRS VOT with clinical indicators of microcirculation failure could tease out this association. Fisher’s Z test showed that there was no significant difference between the two groups at any time point.

### Appendix A.3. NIRS and Length of Stay

ROC curves using reoxygenation slope at presentation or reoxygenation slope at presentation and 60 min following resuscitation to predict ICU length of stay greater than 7 days.

There was no significant correlation between reoxygenation slopes at baseline or 60 min with hospital length of stay.

ROC curves describing the use of reoxygenation slope at presentation and one hour after resuscitation to predict ICU survival. These data suggest that this model predicts ICU survival with moderate sensitivity and specificity. This is not strong, but it indicates a predictive signal.
